# The Registered Children's Nurse

**Published:** 1921-06-04

**Authors:** 


### THE MATRONS' AND SISTERS' DEPARTMENT.

### THE REGISTERED CHILDREN'S NURSE.

A great stand was made by the managers of children's hospitals to ensure that the nurses trained in these establishments should be eligible for registration without general training, and the result was the formation of a supplementary register for children's nurses. It is with some dismay that women who have taken up this branch of nursing heard Dr. Goodall's prognostication that the supplementary registers for children's and fever nurses would in time to come fall into disuse if training in children's and infectious disorders becomes part of every nurse's curriculum.

The women who have had most experience in nursing sick children and in training nurses in this branch of work, that is to say, the matrons and sisters of children's hospitals, are practically unanimous as to the necessity for prolonged training in children's wards—not merely a two or three months' training interspersed with the remainder of the curriculum. There is a great deal more to learn than the actual methods employed for children as distinguished from adults. The nursing of sick children requires quite a long apprenticeship if it is to succeed as it ought to do, and it will be a great misfortune if this specialisation which has been so fruitful for good in the saving of child-life gives place to mere routine study of "children's diseases." The good children's nurse is almost startlingly alive to the slightest change in the bodily and mental sensations of her charges. She has to rely on a sixth sense in divining their needs and it takes some time to acquire it. The best observers maintain that it takes three years to make a probationer really safe with children.

It is quite true that all the best children's hospitals stipulate for general training also in the sisters of their wards. Three years in a children's hospital, three years in a general hospital, six years in all spent in the status of a probationer, represent the apprenticeship deemed necessary in this branch of work. It is a very hard saying. While by no means minimising the degree of experience requisite for the woman who has charge of a ward full of sick children, we would urge that the prospect of being paid and treated as a probationer for six long years is not very encouraging. It ought to be possible so to standardise the training in the two hospitals as to reduce the actual term of training to four years, the probationer enjoying the pay and status of a staff-nurse during any further term she should spend in qualifying herself to become a sister. In this way the probationer training for a sister's post would first receive two years' children's training, then two years' general training, the courses being properly co-ordinated, then two years again in a responsible position at good pay in the children's hospital. But during this later period it would surely be reasonable that facilities should be given her for qualifying as Health Visitor, and for Public Health posts in connection with Infant Welfare work.

The main difficulty about exclusive training in children's hospitals lies in the impracticability of limiting the nurse's work to pure specialism. In this respect statistics are sadly lacking. We do not know, we doubt if any one knows, the extent of the actual demand for nurses trained in the nursing of sick children. What is the position of such a nurse when she begins to take up private work? Can she confine her talents entirely to the line she has chosen? Or would she be exposed to long periods of inaction if she resolved to nurse children and children only? Probably there are half a dozen large co-operations and a few children's doctors able to keep a children's nurse constantly occupied. For the most part we imagine these special nurses find themselves in competition with the women who have fitted in a brief term of children's nursing in the course of their general training. And thus they for their part drift into undertaking cases among adults for which they have received no training whatever.

This is the danger. Four years' training divided between the two training-schools would obviate it.

